# Macrophage colony-stimulating factor (CSF1) controls monocyte production and maturation and the steady-state size of the liver in pigs

**DOI:** 10.1152/ajpgi.00116.2016

**Published:** 2016-07-21

**Authors:** Kristin A. Sauter, Lindsey A. Waddell, Zofia M. Lisowski, Rachel Young, Lucas Lefevre, Gemma M. Davis, Sara M. Clohisey, Mary McCulloch, Elizabeth Magowan, Neil A. Mabbott, Kim M. Summers, David A. Hume

**Affiliations:** ^1^The Roslin Institute and Royal (Dick) School of Veterinary Studies, University of Edinburgh, Easter Bush, Scotland, United Kingdom; and; ^2^Agri-Food and Biosciences Institute, Large Park, Hillsborough, Northern Ireland, United Kingdom

**Keywords:** hepatosplenomegaly, CD163, macrophages, hepatostat, M-CSF

## Abstract

Macrophage colony-stimulating factor (CSF1) is an essential growth and differentiation factor for cells of the macrophage lineage. To explore the role of CSF1 in steady-state control of monocyte production and differentiation and tissue repair, we previously developed a bioactive protein with a longer half-life in circulation by fusing pig CSF1 with the Fc region of pig IgG1a. CSF1-Fc administration to pigs expanded progenitor pools in the marrow and selectively increased monocyte numbers and their expression of the maturation marker CD163. There was a rapid increase in the size of the liver, and extensive proliferation of hepatocytes associated with increased macrophage infiltration. Despite the large influx of macrophages, there was no evidence of liver injury and no increase in circulating liver enzymes. Microarray expression profiling of livers identified increased expression of macrophage markers, i.e., cytokines such as TNF, IL1, and IL6 known to influence hepatocyte proliferation, alongside cell cycle genes. The analysis also revealed selective enrichment of genes associated with portal, as opposed to centrilobular regions, as seen in hepatic regeneration. Combined with earlier data from the mouse, this study supports the existence of a CSF1-dependent feedback loop, linking macrophages of the liver with bone marrow and blood monocytes, to mediate homeostatic control of the size of the liver. The results also provide evidence of safety and efficacy for possible clinical applications of CSF1-Fc.

## Abstract (precis)

*This study is based on extensive studies in the mouse of the role of CSF1 in monocyte-macrophage production and differentiation and the function of macrophages in the control of hepatocyte proliferation. We use a novel form of CSF1, an Fc fusion protein, to demonstrate that the findings in mice can be extended to large animals. We discuss the possible role for CSF1 in homeostatic control of the size of the liver*.

## NEW & NOTEWORTHY

*This study is based on extensive studies in the mouse of the role of CSF1 in monocyte-macrophage production and differentiation and the function of macrophages in the control of hepatocyte proliferation. We use a novel form of CSF1, an Fc fusion protein, to demonstrate that the findings in mice can be extended to large animals. We discuss the possible role for CSF1 in homeostatic control of the size of the liver*.

macrophage colony-stimulating factor (CSF1) is an essential growth and differentiation factor for cells of the macrophage lineage (23). Subsequent to the original isolation of human CSF1 cDNA in the 1980s and demonstration that injection of the recombinant CSF1 protein can expand macrophage populations in mice (24), there were a number of clinical trials of applications in cancer and other indications (23). The interest in CSF1 as a therapeutic agent has been reinvigorated by evidence of the requirement for macrophages in tissue regeneration in multiple organs (7) and the finding that macrophages generated in response to CSF1 have trophic roles (9, 43). CSF1 treatment has been shown to promote regeneration and repair in injury models in the kidney (1), brain (5, 37), and bone (6). The pleiotropic impacts of CSF1 mutations in mice (12) suggest that repair in most tissues is reliant on macrophages that depend on this growth factor.

Applications of CSF1 therapy were constrained by the very short half-life of the 150-amino acid active form of CSF1. We developed a bioactive protein with a longer half-life in the circulation. We fused pig CSF1, which is equally active in all mammalian species tested (20), with the Fc region of pig IgG1a (CSF1-Fc) (21). The expected increase in half-life was confirmed, and CSF1-Fc administration to mice produced substantial increases in circulating monocyte and tissue macrophage numbers, at much lower doses than the native protein. An unexpected effect of the treatment was a substantial increase in the size of the liver, associated with extensive hepatocyte proliferation (21). This observation was consistent with previous data implicating CSF1-dependent macrophages in hepatic regeneration. Acute liver failure in human patients, for example due to paracetamol toxicity, is associated with the loss of clearance functions that protect the body against the contents of the portal blood. Circulating CSF1 levels in human patients upon admission to hospital with paracetamol poisoning were found to be predictive of subsequent prognosis (46). Administration of CSF1-Fc in mouse models produced both accelerated regeneration of the liver and, perhaps more importantly, very rapid restoration of clearance functions (21, 44, 46).

Pigs have been used increasingly as models of human disease (17). The gene expression profiles of stimulated mouse and human macrophages differ greatly, and those of pig macrophages are much more human-like (29). Aside from the possible applications as human disease models, pigs are a major livestock species. Early weaning in pigs, when the mucosal barrier and innate immune systems are immature, is associated with susceptibility to a very wide range of mucosal and systemic bacterial and viral infections that produce significant losses (3). The results obtained in mice cannot necessarily be extrapolated to large animals, including humans, in which the profiles of macrophage activation by both CSF1 and microbial stimuli are very different (29). Our results confirm that, as in the mouse, CSF1-Fc can drive hepatocyte proliferation and modulate the size of the liver in a large animal model. The data support applications of CSF1-Fc in liver regeneration and provide further evidence for the role of CSF1 in monocyte/macrophage maturation. In combination with earlier data, they reinforce the conclusion that circulating CSF1 is a central contributor to the homeostatic control of the size of the liver.

## MATERIALS AND METHODS

### 

#### Animals.

Approval was obtained from Protocols and Ethics Committees of Roslin Institute or Agri-Food and Biosciences Institute (AFBI) for the trials. The experiments were carried out under the authority of a UK Home Office Project License, under the regulations of Animals (scientific procedures) Act 1986. CSF1-Fc was made as previously described (21) and provided by Zoetis.

Large White pigs ∼8.5 wk of age from one litter were used. Four days prior to the first injection each pig was weighed and had blood collected into an EDTA tube. Pigs were injected subcutaneously once a day for a total of 3 days with the appropriate volume of CSF1-Fc (0.75 mg/kg; *n* = 6) or PBS vehicle (*n* = 5). PBS injection was used to control for the possible impact of stress and restraint associated with treatment. In experiments on weaners, Large White × Landrace pigs ∼4 wk of age from three litters were used. Six days prior to the first injection each pig was weighed and an estimated weight was extrapolated for each for the first injection day. Pigs were injected intramuscularly once a day for 2 days with the appropriate volume of CSF1-Fc (0.75 mg/kg; *n* = 12) or PBS (*n* = 12). On the second injection day the pigs were weaned. All pigs were sedated with ketamine and azaperone before being euthanized by captive bolt. Neither subcutaneous nor intramuscular injection produced any side effects.

#### Isolation of PBMC and BMC.

Blood was collected into blood collection bags containing acid citrate dextrose (ACD) (Sarstedt) or into beakers containing ACD (Sigma). The buffy coat was layered onto Lymphoprep (Axis-Shield) and centrifuged for 25 min at 1,200 *g* with no brake. Peripheral blood mononuclear cells (PBMC) were retrieved and red cells were removed with cell lysis buffer (BioLegend). Pig bone marrow cells (BMC) were obtained by flushing the bone marrow from ribs with RPMI/5 mM EDTA followed by removal of red cells with cell lysis buffer. All isolated cells were suspended in PBS prior to counting and cryopreservation.

#### Flow cytometry analysis.

Cells were washed, pelleted, resuspended in blocking buffer (PBS/2% heat inactivated FCS), transferred to a 96-well plate (V-bottom), and incubated on ice for 15–20 min. The plate was centrifuged for 4 min at 400 *g* followed by removal of supernatant. Cells were resuspended in 100 μl of PBS containing the appropriate antibody or isotype control (Table 1). Samples were incubated at 4°C in the dark for 30 min before being washed two times with 200 μl PBS. Cells were resuspended in 600 μl PBS with 0.1% SYTOX blue (Invitrogen) immediately prior to analysis using a BD Fortessa LSR flow cytometer (Becton Dickinson). Analysis was performed using FlowJo software (FlowJo).

**Table 1.** Antibodies used in flow cytometry

Antigen	Conjugate	Isotype	Supplier	Dilution	Isotype Control
CD16	RPE	IgG1	AbD Serotec, MCA1971PE	1:200	AbD Serotec, MCA928PE
CD14	FITC	IgG2b	AbD Serotec, MCA1218F	1:50	AbD Serotec, MCA691F
CD163	RPE	IgG1	AbD Serotec, MCA2311PE	1:100	AbD Serotec, MCA928PE
CD172a	RPE	IgG1	Southern Biotech, 4525-09	1:400	AbD Serotec, MCA928PE
CD169	FITC	IgG1	AbD Serotec, MCA2316F	1:100	AbD Serotec, MCA928F
CD117	AF488	IgG1	AbD Serotec, MCA2598A48	1:10	Biolegend, 400109
CD3	FITC	IgG1	AbD Serotec, MCA5951F	1:100	AbD Serotec, MCA928F

Antigen	Conjugate	Isotype	Supplier	Concentration	Secondary Antibody
CSF1R	Purified	IgG2a	Produced in-house	5 μg/ml	Biolegend, 405308, 1:400 dilution

Summary information for all monoclonal antibodies used for flow cytometry staining, including dilution, manufacturer, and relevant isotype control.

#### Complete blood count analysis.

An aliquot of blood from ACD blood collection bags was analyzed for complete blood cell counts. Total white blood cell (WBC) was measured on the Siemens Advia 2120 analyzer. WBC differential counts were performed by making a blood smear counterstained with Giemsa stain prior to cells of each cell type being counted. The absolute value for each WBC type was determined by using the total WBC and % leukocytes. Manual platelets counts were carried out using a hemocytometer slide (by the R(D)SVS Clinical Pathology Laboratory, University of Edinburgh).

#### Plasma analysis.

Plasma was analyzed by R(D)SVS Clinical Pathology Laboratory for cortisol (performed on Siemens Immulite analyzer) as well as a large animal liver damage profile (performed on the IL650 analyzer from Instrumentation Laboratories).

#### Tissue processing.

Tissues were dissected, weighed (liver, spleen, and kidney), and placed in 10% neutral buffered formalin or RNAlater (Ambion). For histology, tissues were processed overnight using an Excelsior tissue processor (Thermo Fisher Scientific). Sections were embedded in paraffin wax prior to 4-μm sections cut and mounted onto slides (Superfrost Plus, Thermo Fisher Scientific). Slides were dried overnight at 37°C before 60°C for 25 min. Sections were stained with H&E or immunohistochemistry was performed by R(D)SVS pathology department.

#### Immunohistochemistry for CD163.

Antigen retrieval was performed with proteinase K (Dako S302030) for 10 min. Nonspecific protein binding was blocked using 2.5% goat serum (Vector Laboratories) for 20 min. Endogenous peroxidase activity was blocked using Dako REAL peroxidase blocker (Dako S202386) for 10 min. Sections were incubated for 60 min using mouse anti-pig CD163 (Serotec MCA2311GA) diluted 1/30. Visualization using secondary reagent Dako Envision mouse HRP (Dako K4007) for 40 min followed by DAB (Newmarket Scientific Monosan Dab substrate kit cat. no. MON-APP177) for 10 min and DAB enhancer for 3 min (Newmarket Scientific DAB concentrate cat. no. CO7-25) was performed by the R(D)SVS pathology department. The staining was analyzed using Image J (Fiji).

#### Immunohistochemistry for Ki67 and PCNA.

Antigen retrieval was performed by boiling in 10 mM sodium citrate buffer. Nonspecific protein binding was blocked using 2.5% goat serum (Vector Laboratories) for 20 min. Endogenous peroxidase activity was blocked using Dako REAL peroxidase blocker (Dako S202386) for 10 min. Sections were incubated for 60 min using rabbit anti-human Ki67 (AbCam AB15580) diluted 1/10,000. Visualization was performed with secondary reagent ImmPRESS HRP anti-rabbit IgG (Peroxidase Polymer; VectorMP-7401) for 30 min followed by DAB (Newmarket Scientific Monosan Dab substrate kit cat. no. MON-APP177) for 10 min and DAB enhancer for 3 min (Newmarket Scientific DAB concentrate cat. no. CO7-25).

#### Statistical analysis.

Data were analyzed by *t*-tests. Results are presented as treatment group means ± SE. All analyses were performed using GraphPad Prism 5.0 (GraphPad Software). A *P* value < 0.05 was considered statistically significant.

#### Microarray.

Total RNA was prepared from liver samples using TRIzol, prepared for hybridization using the Ambion WT Expression Kit (Life Technologies), following the manufacturer's instructions, except for the input amount of RNA (500 ng input instead of 100 ng) and hybridized in a random order to the Affymetrix Porcine Gene 1.1 ST array (performed by Edinburgh Genomics, University of Edinburgh). Statistical analysis of the array data utilized Partek Genomic Suite (Partek). For network analysis, the normalized array data were uploaded to the software Biolayout *Express*^3D^ (http://www.biolayout.org/) as described previously (18, 30). The data from the microarray are available at Gene Expression Omnibus NCBI (http://www.ncbi.nlm.nih.gov/geo/) accession code GSE78837.

## RESULTS

### 

#### CSF1-Fc expands macrophage populations in blood and organs.

**Fig. 1.**
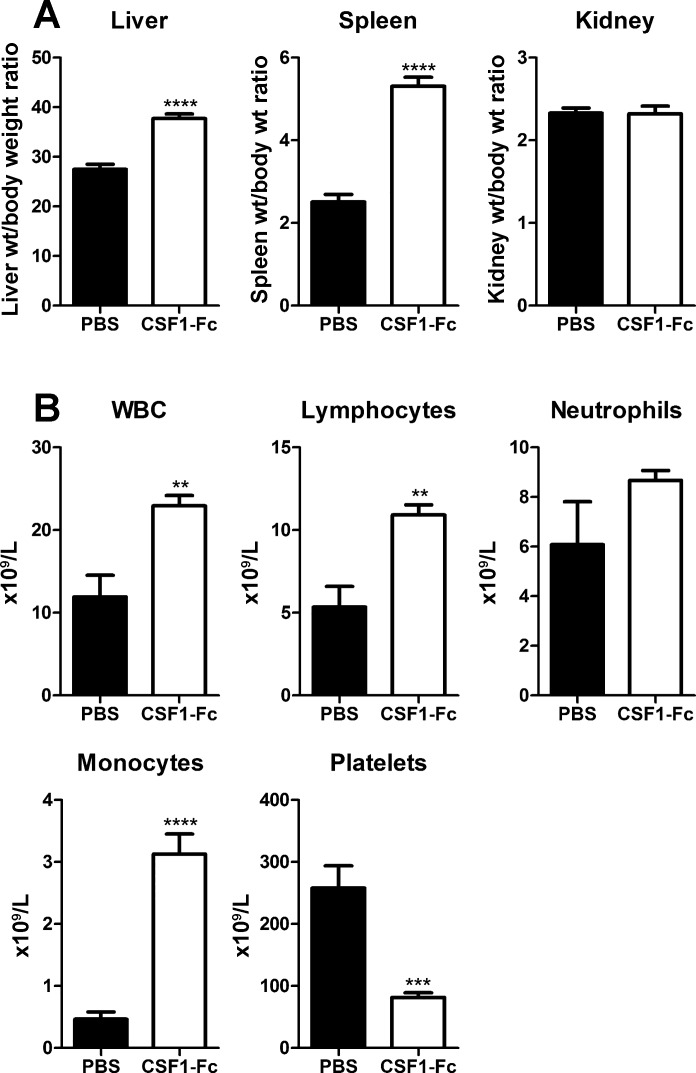
Effect of CSF1-Fc on organ weights and white blood cell (WBC) counts. Pigs (8-wk-old males and females) were injected with PBS or 0.75 mg/kg CSF1-Fc for 3 days prior to euthanasia on *day 4*. Blood was collected into EDTA tubes postmortem and complete blood count assessment was performed. Graphs show means ± SE. **P < 0.01, ***P < 0.001, ****P < 0.0001 by *t*-test; *n* = 5–6 pigs per treatment. *A*: liver weight/body weight ratio, spleen weight/body weight ratio, and kidney weight/body weight ratio. *B*: total WBC count, lymphocyte number, neutrophil number, monocyte number, and platelet number.

We first examined 8-wk-old pigs of both sexes from the same litter, with control and treated groups weight matched in pairs. Based on mouse data (21) we used a dose of 0.75 mg/kg for three daily treatments followed by cull 24 h after final injection. In previous studies, we have compared CSF1-Fc with the native, non-Fc conjugate, form of CSF1 as a control protein. Native CSF1 has a much shorter half-life and at the same dose had no effect on monocyte-macrophage numbers (21). We have not compared CSF1-Fc directly with an irrelevant fusion protein, or with an isotype control for the Fc component. However, an equivalent dose of IgG1 (10–20 mg depending on the size of the pig) would have no impact on the plasma IgG concentration (15–30 mg/ml). CSF1-Fc treatment of macrophages in vitro did not induce proinflammatory cytokines (21) and, in keeping with the lack of intrinsic proinflammatory activity, there was no evidence of any reaction at the sites of injection in any treated animals. The most obvious effect of CSF1-Fc administration was hepatosplenomegaly. CSF1-Fc doubled the spleen/body weight ratio and increased the liver/body weight ratio by 40% after only 4 days (Fig. 1*A*). The total WBC count was significantly increased, mainly due to lymphocytosis in addition to the expected monocytosis (Fig. 1*B*).

#### CSF1-Fc accelerates the maturation of macrophage populations in peripheral blood monocytes.

**Fig. 2.**
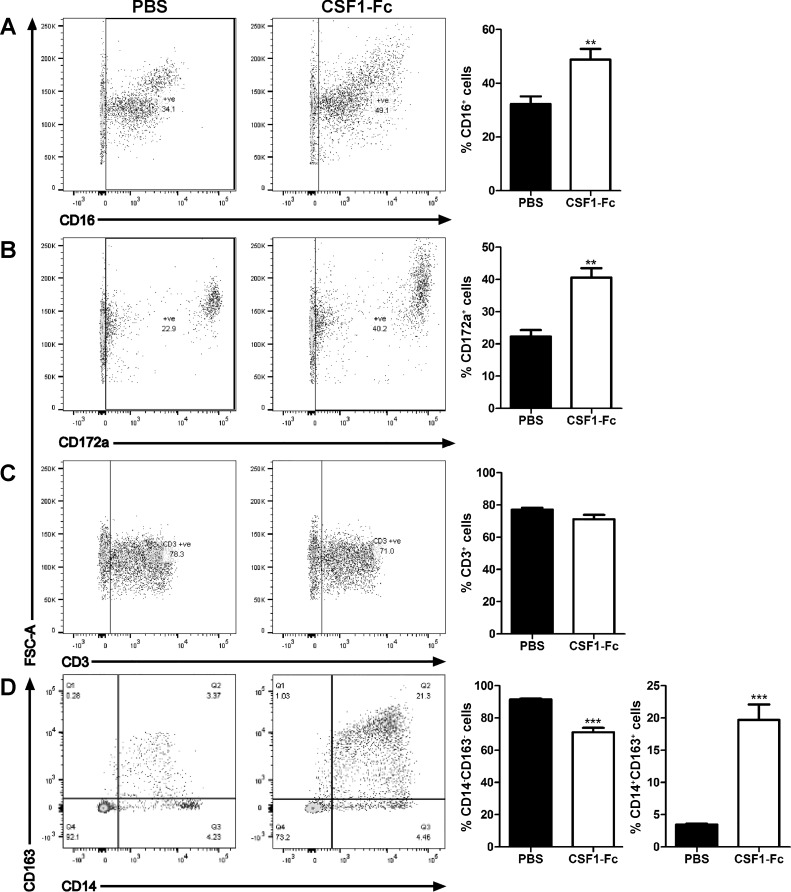
Effect of CSF1-Fc on PBMCs. Pigs (8-wk-old males and females) were injected with PBS or 0.75 mg/kg CSF1-Fc for 3 days prior to euthanasia on *day 4*. Blood was collected as described in materials and methods. PBMCs were analyzed via flow cytometry for expression of CD16 (*A*), CD172a (*B*), CD3 gated on the lymphocyte population exclusively (*C*), or CD163/CD14 (*D*) with exclusion of dead cells using SYTOX blue. Representative flow cytometry plots are shown. Graphs show means ± SE. **P < 0.01, ***P < 0.001 by *t*-test; *n* = 4–5 pigs per treatment.

CSF1 has been implicated in the maturation of blood monocytes in both mice and humans, driving the formation of the nonclassical (CD14^low^, CD16^high^) subset in humans (Ly6C^low^ in mice) (31, 33). Pig blood monocytes can also be separated into subsets based on expression of surface markers, although the distinctions are not as clear as in other species (16). Expression of various markers by peripheral blood monocytes was assessed by flow cytometry staining (Fig. 2). In addition to the increase in total WBC seen in Fig. 1, the proportion of monocytes, detected by CD172a (SIRPA) was increased around twofold (Fig. 2*B*). The proportion of cells expressing CD16 was also increased (Fig. 2*A*). No increase was seen in the percentage of CD3^+^ lymphocytes (Fig. 2*C*) The best-characterized monocyte maturation marker in pigs is the haptoglobin receptor, CD163 (16), which has also been implicated as a receptor for the major pig viral pathogen, porcine reproductive and respiratory syndrome (PRRSV) (14), and which varies inversely with CD14 expression. Double staining with the two markers indicated that CSF1-Fc shifted the profiles of both markers, favoring expansion of the CD163^+^ cells (Fig. 2*D*). The results are consistent with an impact of CSF1-Fc to promote both monocyte production/release and maturation.

#### Impact of CSF1-Fc treatment on the bone marrow.

**Fig. 3.**
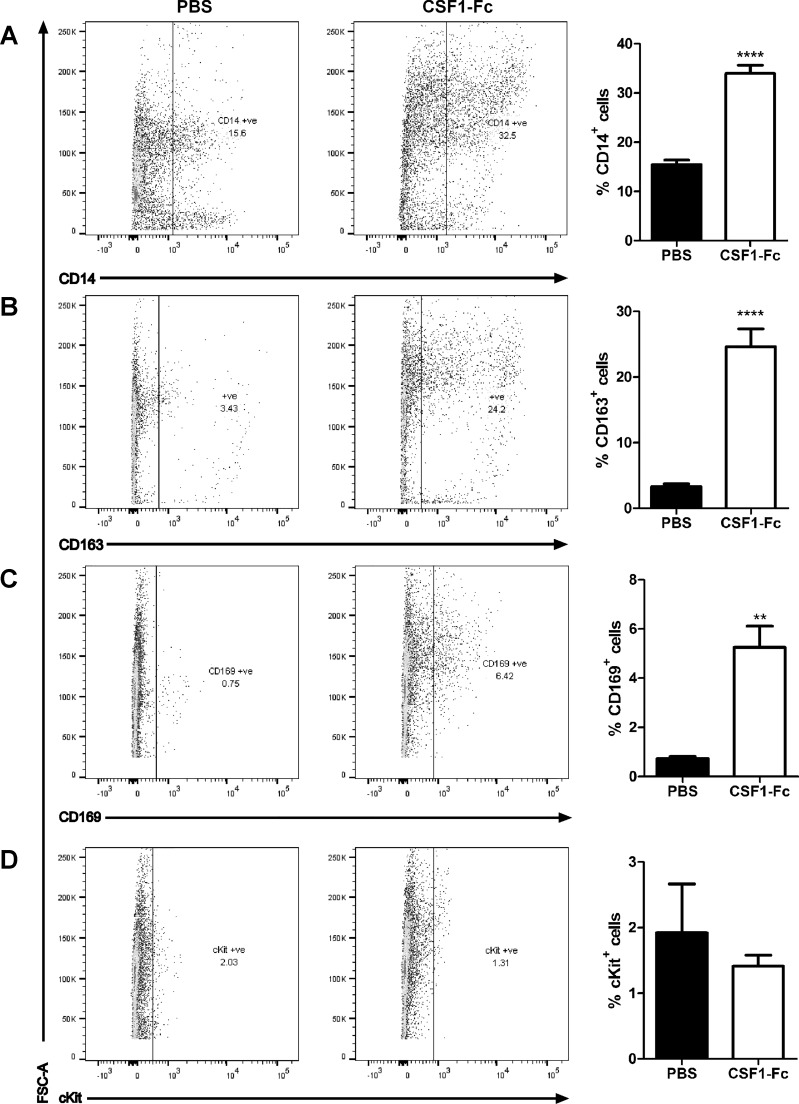
Effect of CSF1-Fc on bone marrow (BM) cells. Pigs (8-wk-old males and females) were injected with PBS or 0.75 mg/kg CSF1-Fc for 3 days prior to euthanasia on *day 4*. BM from ribs was collected as described in materials and methods. BM cells were analyzed via flow cytometry for expression of CD14 (*A*), CD163 (*B*), CD169 (*C*), or cKit (*D*) with exclusion of dead cells using SYTOX blue. Representative flow cytometry plots are shown. Graphs show means ± SE. **P < 0.01, ****P < 0.0001 by *t*-test; *n* = 4–5 pigs per treatment.

**Fig. 4.**
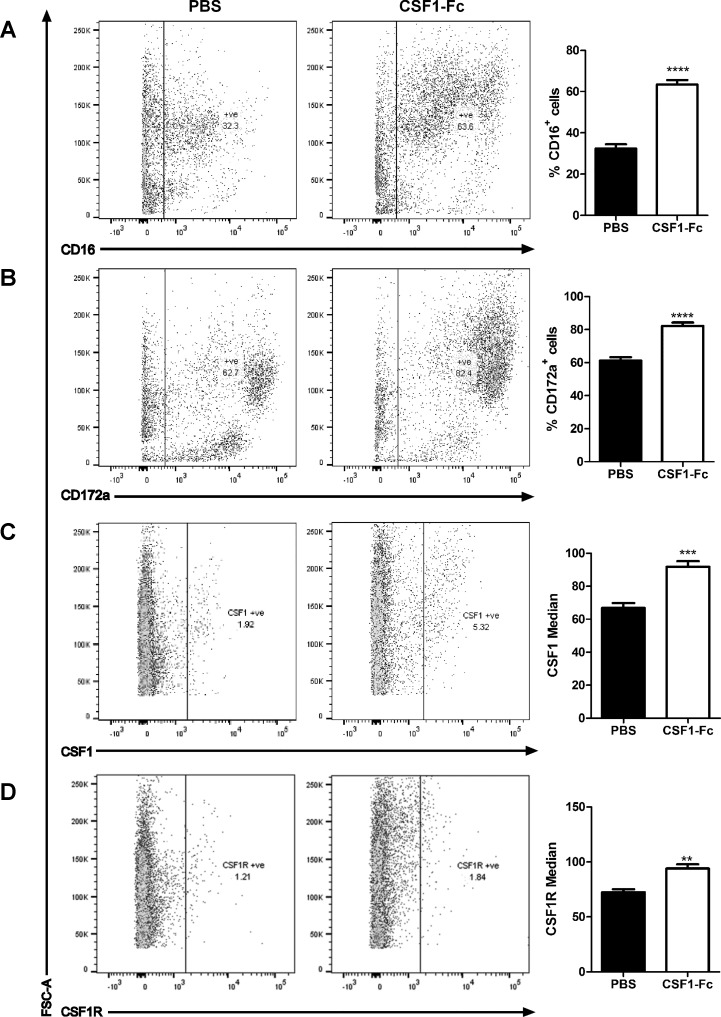
Further effect of CSF1-Fc on BM cells. Pigs (8-wk-old males and females) were injected with PBS or 0.75 mg/kg CSF1-Fc for 3 days prior to euthanasia on *day 4*. BM from ribs was collected as described in materials and methods. BM cells were analyzed via flow cytometry for expression of CD16 (*A*), CD172a (*B*), CSF1 (*C*), or CSF1R (*D*) with exclusion of dead cells using SYTOX blue. Representative flow cytometry plots are shown. Graphs show the mean percentage positive cells ± SE, or the median fluorescent intensity. **P < 0.01, ***P < 0.001, ****P < 0.0001 by *t*-test; *n* = 4–5 pigs per treatment.

We next investigated whether the ability of CSF1-Fc to promote monocytosis was associated with expansion of progenitor pools in the marrow (Fig. 3). Figure 3, *A* and *B*, demonstrates a substantial increase in large CD14^+^ monocytes, and even greater increase in CD163^+^ cells in the marrow of treated animals, consistent with the pattern seen in blood. Aside from monocyte progenitors, a key population of macrophages in bone marrow forms the center of hemopoietic islands. In mice, these cells express sialoadhesin (CD169, which provides a receptor for immature erythrocytes) and are critical for successful engraftment in bone marrow transplantation (10). As shown in Fig. 3*C*, the CSF1-Fc treatment produced a substantial increase in the CD169^+^ population in pigs. The CSF1-Fc treatment did not expand the small percentage of cells that express CD117 (KIT), a marker of the stem cell population (Fig. 3*D*), suggesting that CSF1-Fc acts primarily to promote proliferation/expansion of committed progenitors. In bone marrow of pig, neither CD172a nor CD16 provides a useful marker of monocyte lineage cells, being detected on the large majority of the cells (Fig. 4, *A* and *B*) and only marginally increased by CSF1-Fc. We also examined the expression of the CSF1R (CD115) using either a recently described monoclonal antibody (39) or labeled CSF1-Fc. There was some evidence of expansion of the positive cell populations in each case, but the levels of labeling were very low (Fig. 4, *C* and *D*). We suggest that the receptor may be downregulated by CSF1-Fc.

#### Origin of the increase in liver and spleen weight.

**Fig. 5.**
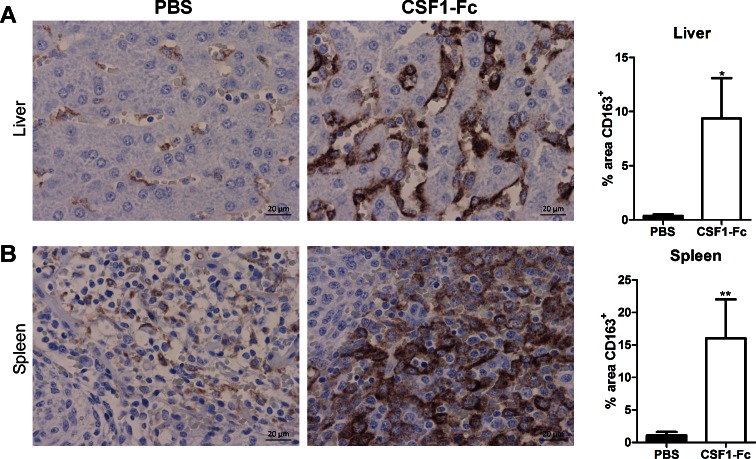
Effect of CSF1-Fc on tissue macrophages. Pigs (8-wk-old males and females) were injected with PBS or 0.75 mg/kg CSF1-Fc for 3 days prior to euthanasia on *day 4*. Formalin fixed liver (*A*) and spleen (*B*) tissue was prepared and stained for CD163. Representative images are shown. Scale bar = 20 μM. Using ImageJ software the total CD163^+^ area was calculated from 2 representative images per pig per organ. Graphs show means ± SE. *P < 0.05, **P < 0.01 by *t*-test; *n* = 4–5 pigs per treatment.

CSF1-Fc treatment caused a substantial increase in macrophage numbers in both organs, detectable by immunolocalization of CD163. In immunostained sections of liver CSF1-Fc treatment increased CD163^+^ area (quantified with ImageJ) from an average of less than 0.5% to an average of over 9% (Fig. 5*A*). In spleen CSF1-Fc treatment had an even greater effect, causing an increase of CD163^+^ area from an average from ∼1% to 16% (Fig. 5*B*). As in mice (21), in the spleen the majority of the increase in size could be attributed to increased red pulp macrophages and also to expansion of the marginal zones.

**Fig. 6.**
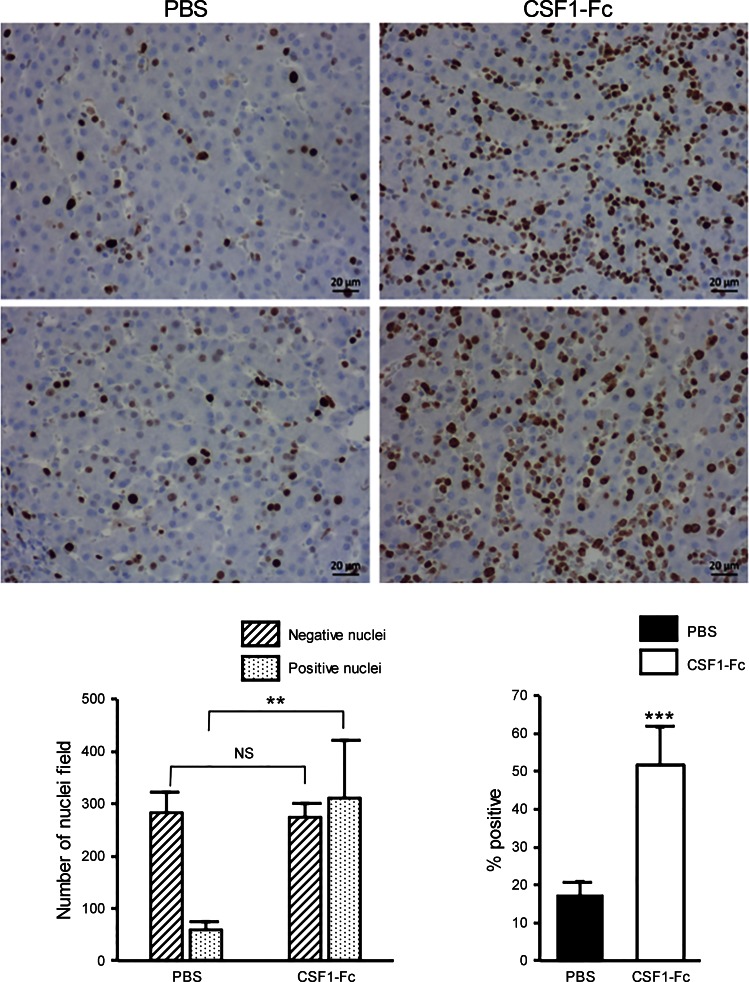
Effect of CSF1-Fc on liver proliferation. Pigs (8-wk-old males and females) were injected with PBS or 0.75 mg/kg CSF1-Fc for 3 days prior to euthanasia on *day 4*. Formalin fixed liver tissue was prepared and stained for Ki67 as described in materials and methods. *Top*: representative fields from 2 separate control (PBS, *left*) or CSF1-Fc-treated (*right*) pigs. Individual Ki67^+^ and Ki67^−^ nuclei in representative fields from each animal were counted as shown at *bottom*. **P < 0.01, ***P < 0.001; NS, not significant.

By contrast, the increase in the area apparently occupied by macrophages is not sufficient to explain the substantial increase in the size of the liver. Sections of liver were stained for the proliferative cell marker Ki67. Figure 6 shows images of the liver from two control and two CSF1-Fc-treated pigs. The pigs are relatively young, and still growing, and accordingly there is significant ongoing proliferation evident from Ki67 staining. The vast majority of Ki67^+^ nuclei in both control and CSF-1-Fc-treated pig livers were large and round, consistent with identity as hepatocytes. Macrophage nuclei are more difficult to visualize in histological sections, because the cells and nuclei are much smaller and ramified in the sinusoids. Very occasional smaller Ki67^+^ nuclei visible in the sinusoids suggested that some infiltrating monocyte-macrophages were also proliferative, as shown directly in the mouse system (46). The images in Fig. 6 also show an obvious increase in cellularity in response to CSF1-Fc. We counted the total nuclei and the proportion stained with anti-Ki67 in representative large fields from each animal. As shown in Fig. 6, CSF1-Fc treatment almost doubled the total number of nuclei in each field and produced a threefold increase in the percentage of those nuclei stained with anti-Ki67. Essentially the same findings were made with staining for proliferating cell nuclear antigen (PCNA) (not shown). The sections in Fig. 6 show no evidence of pathology in the liver; notably, there are no pyknotic nuclei and granulocytes are absent. Granulocyte infiltration is the hallmark of tissue injury, including injury to the liver (27).

**Fig. 7.**
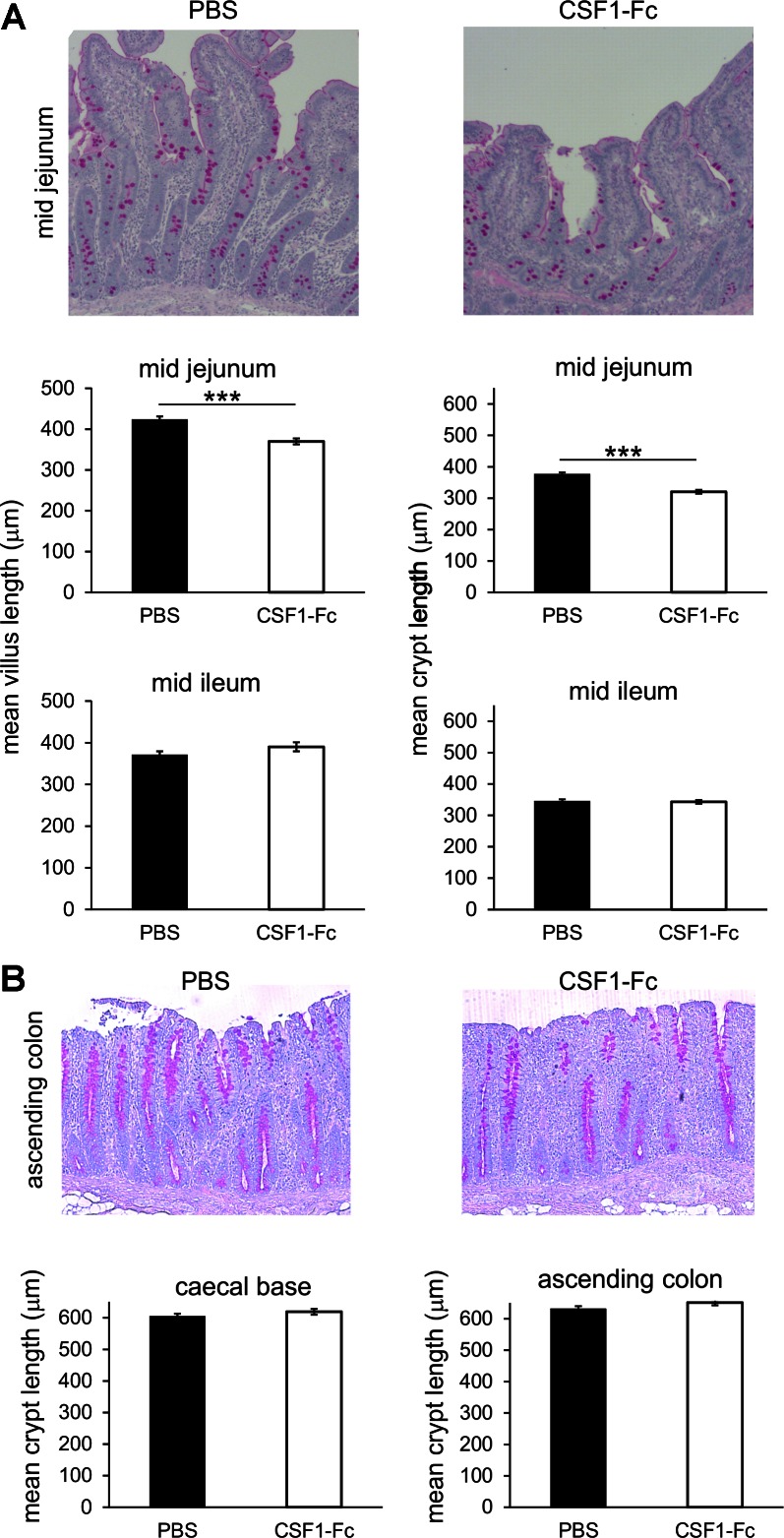
Effect of CSF1-Fc on the gut. Pigs (8-wk-old males and females) were injected with PBS or 0.75 mg/kg CSF1-Fc for 3 days prior to euthanasia on *day 4*. Formalin fixed sections of gut tissue were prepared and stained for periodic acid-Schiff to show the goblet cells. Representative images are shown. Graphs show means ± SE. ***P < 0.001 by *t*-test; *n* = 4–5 pigs per treatment. *A*: small intestine: mid jejunum and mid ileum. Mean villus length (μm) was measured from 112–120 villi per tissue per group. Mean crypt length (μm) was measured from 100 crypts per tissue per group. *B*: large intestine: ascending colon and cecal base. Mean crypt length (μm) was measured from 50–80 crypts per tissue per group.

Changes in the liver might occur secondary to alterations in the gut. CSF1 has been attributed indirect functions in control of proliferation and differentiation of gastrointestinal epithelium (26, 45). Treatment with CSF1-Fc in pigs produced a small but significant increase in the mean villus length of the mid jejunum but had no detectable effect in the ileum, cecal base, or ascending colon (Fig. 7). There was also no significant change in goblet cell number.

#### Effect of CSF1-Fc on liver function.

**Fig. 8.**
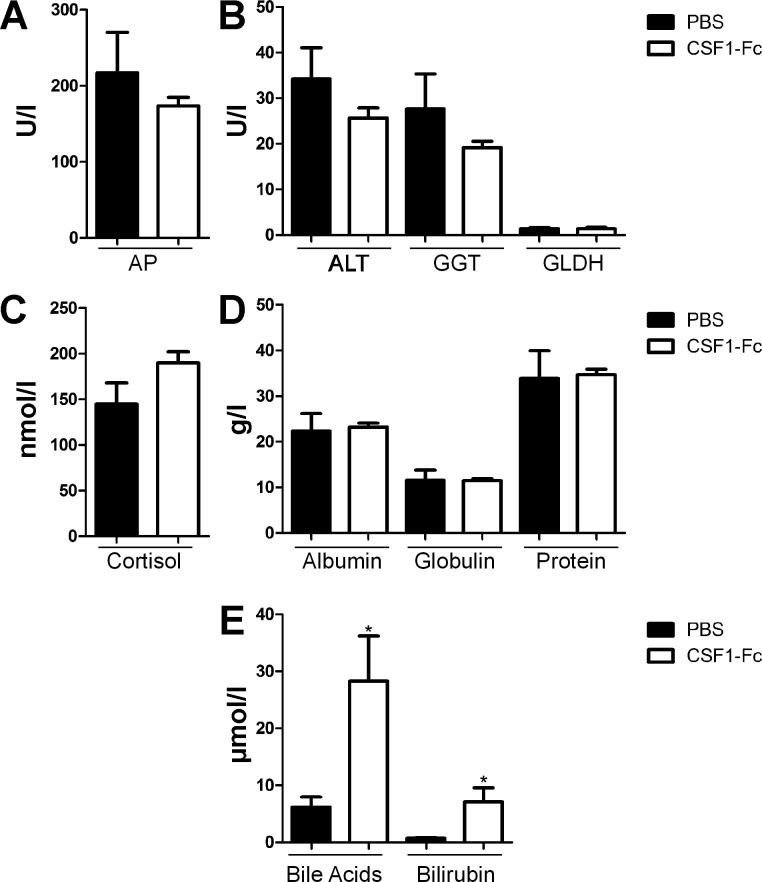
Effect of CSF1-Fc on circulating markers of liver damage. Pigs (8-wk-old males and females) were injected with PBS or 0.75 mg/kg CSF1-Fc for 3 days prior to euthanasia on *day 4*. Plasma was produced from whole blood collected in an ACD blood collection bag. Graphs show means ± SE. *P < 0.05 by *t*-test; *n* = 5–6 pigs per treatment. *A*: alkaline phosphatase (AP). *B*: alanine aminotransferase (ALT), γ-glutamyl transpeptidase (GGT), and glutamate dehydrogenase (GLDH). *C*: cortisol. *D*: albumin, globulin, and total protein. *E*: bile acids, and bilirubin.

**Fig. 9.**
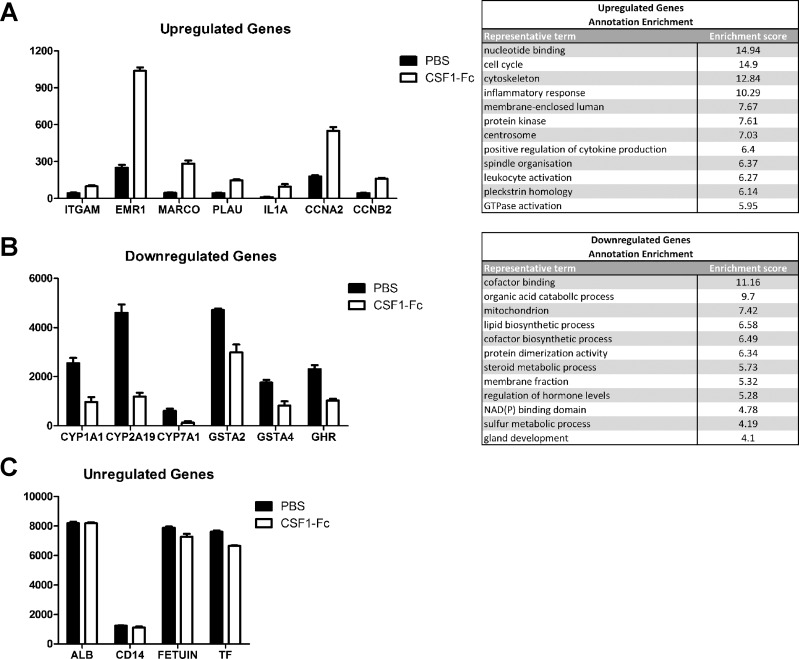
Effect of CSF1-Fc on gene expression in the liver. Pigs (8-wk-old males and females) were injected with PBS or 0.75 mg/kg CSF1-Fc for 3 days prior to euthanasia on *day 4*. Liver tissue was collected in RNAlater and RNA was prepared and submitted for microarray analysis. *A*: expression profiles of a number of genes from *cluster 1*: upregulated genes in CSF1-Fc-treated pigs and a table of the top 12 enrichment clusters with representative terms. *B*: expression profiles of a number of genes from *cluster 2*: downregulated genes in CSF1-Fc-treated pigs and a table of the top 12 enrichment clusters with representative terms. *C*: expression profiles of a number of genes that were unregulated.

A panel of biochemical tests to measure serum enzymes, bile acid, bilirubin, and protein concentrations was performed to assess hepatic function. The only change was a small increase in bile acids and bilirubin in serum from CSF1-Fc-treated pigs (Fig. 8), only marginally outside the normal range (50). Since standard enzymic indicators of liver injury (alkaline phosphatase, alanine aminotransferase, γ-glutamyl transpeptidase) were unchanged, the increase in bile acids probably reflects the increased size of the liver. To examine the impact of CSF1-Fc on liver function in more detail, we profiled the transcriptome. The expression results were filtered to remove genes detected below an arbitrary relative intensity threshold and also genes that did not differ by more than 1.5-fold between the highest and lowest value in the nine samples. The second criterion removed around 30% of probes on the microarray, including many hepatocyte-specific gene products. Figure 9*C* shows that the relative abundance of representative examples of these known hepatocyte gene products, albumin (*ALB*), *CD14*, *FETUIN*, and transferrin (*TF*), within the total liver RNA pool was unchanged in response to CSF1-Fc. In other words, the infiltration of the liver by macrophages was insufficient to dilute the contribution of hepatocyte mRNA to the total mRNA pool. That finding is consistent with the histological observation above, that even in the CSF1-Fc-stimulated state the infiltrating macrophages appear to make up no more than 10% of the total area of the liver. Hence, the 40% increase in total liver weight can be attributed primarily to an increase in hepatocytes, consistent with the extensive proliferation and increased cellularity shown in Fig. 6.

We clustered the included probe sets based on expression pattern and displayed them using Biolayout *Express*^3D^. The advantage of using the clustering approach is that genes that might appear regulated, but in only a subset of animals, appeared in separate smaller clusters. These may reflect the interanimal variation in macrophage-inducible gene expression that we documented previously in a study of pig breeds (30). The gene lists of specific clusters are provided in Supplemental Table S1. (Supplemental Material for this article is available online at the Journal website.) Functional annotations of the two large clusters were tested using DAVID (Supplemental Table S2). *Cluster 1*, the set of genes elevated in all CSF1-Fc-treated pigs, was clearly enriched for genes involved in the cell cycle and innate immunity, whereas *cluster 2*, the set that was reduced in the CSF1-Fc-treated pigs, was enriched in genes involved in metabolism. Importantly, there is no evidence among the induced genes of expression of apoptosis-associated genes, no induction of acute phase genes, and no appearance of classical granulocyte marker genes such as S100A8/S100A9 or MPO. Figure 9 shows the expression profiles of a number of genes that highlight the biological processes involved. *Cluster 1* (Fig. 9*A*) includes many genes that were shown previously (18) to be restricted to macrophages, such as the transcription factor *SPI1*; surface markers *SIRPA*, *EMR1*, and *ITGAM*; known CSF1-inducible genes *PLAU*, *MMP9*, *CHI3L1*, and *C1Q*; endocytic receptors *MARCO*, *MSR1*, and *FCGR1A*; and pattern recognition receptors *TLR1*, *2*, *4*, *6*, *7*, *8*, and *9*. On average, the relative contribution of *cluster 1*, macrophage-specific genes, to the liver total RNA increased by three- to fivefold in response to CSF1-Fc, again consistent with the histological evidence of increased macrophage content shown in Fig. 5. In mice, the recruited monocytes express the chemokine receptor CCR2 and apparently respond to CCL2 (49). By contrast, *CCR1* was enriched in the liver mRNA of treated pigs, alongside three of its known ligands, *CCL8*, *CCL14*, and *CCL3L1*. As previously observed in mice (46), monocytes recruited to the treated livers apparently responded to proinflammatory signals, since *cluster 1* contained numerous known LPS-inducible genes (29) such as inflammatory cytokines *TNF*, *IL1A*, and *IL1B*; interferon targets *IDO1*; multiple type 1 interferon targets *IRF1*, *IRF5*, *IFITM2*, and *IFIT3*; TGFB1; and costimulators of T cell activation *CD40*, *CD80*, and *CD86*. *Cluster 1* also contains numerous cell cycle-associated genes, including *PCNA*; the key transcription factors *FOXM1*, *E2F4*, *E2F7*, and *E2F8*; and several cyclin genes *CCNA2*, *CCNA3*, *CCNB2*, *CCNB3*, *CCND2*, and *CCND3*. The increased expression of enzymes of glycolysis *HK1*, *HK2*, *HK3 PFKP*, *PGK1*, *PGD*, *PKM*, *GPI*, *GAPDH*, and *LDHA* also reflects the requirement for aerobic glycolysis in proliferating cells (25).

*Cluster 2* (Fig. 9*B*), the set of genes reduced in the CSF1-Fc-treated pigs, most likely reflects the functional zonation of the liver between periportal and perivenous regions of liver lobules (8, 19, 48) and the selective proliferation of cells derived from portal progenitors that has been observed in regenerating liver (15, 34, 36). It includes genes involved in xenobiotic metabolism and detoxification, notably P450 family (e.g., *CYP1A1*, *CYP2E1*), glutathione *S*-transferases (e.g., *GSTAA2*) and aldo-ketoreductases (e.g., *AKR1C1*), and the gluconeogenic enzyme *PCK2*, that are known to be enriched in perivenous locations. The cluster contains the gene for the regulator of hepatocyte stem cells, SOX9 (2), indicating that these cells are not expanded in the CSF1-Fc-treated livers. Unexpected members of this cluster are genes for the growth hormone receptor (*GHR*) and the target, *IGF1*, and both estrogen (ESR1) and androgen (AR) receptors. Also unexpected is the inclusion of the receptor for hepatocyte growth factor, MET, which is implicated in regeneration (36), but this might reflect autoregulation in response to its ligand (52).

#### CSF1-Fc treatment in weaning pigs.

Although models of acute liver failure in pigs have been described (32, 41), and may be one path to clinical development of CSF1-Fc as a treatment, it is challenging to perform sufficient replicates to test a clinical intervention. We therefore considered an alternative in production pigs. Commercial pigs are normally weaned at 4 wk, when the gut is immature. Diarrhea and disseminated infections with organisms such as *Escherichia coli* and *Streptococcus suis* are relatively common (38). In this respect, the pig has been studied as a model of early-life stress (42). The biology of early weaning in pigs may also be relevant to intestinal failure-associated liver disease in neonates and children (40).

**Fig. 10.**
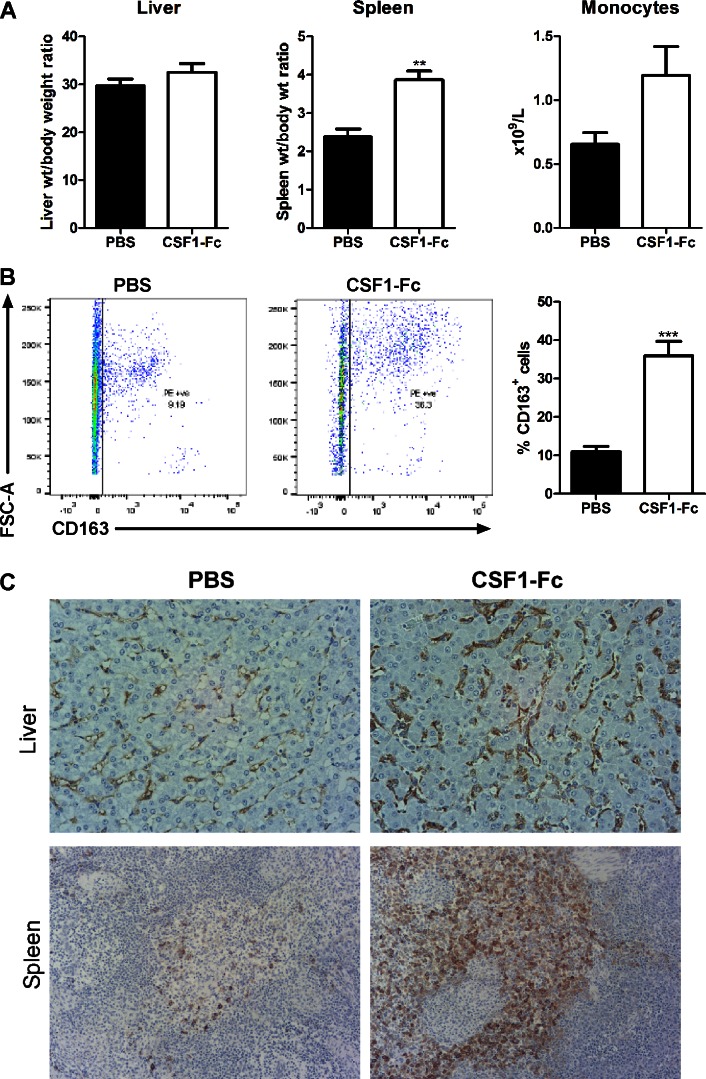
Effect of a short course of CSF1-Fc in weaning pigs. Pigs (4-wk-old males and females) were injected with PBS or 1 mg/kg CSF1-Fc for 2 days prior to euthanasia on *day 3*. Graphs show means ± SE. *P < 0.05, **P < 0.01, ***P < 0.001 by *t*-test; *n* = 5 pigs per treatment. *A*: blood was collected into EDTA tubes postmortem and complete blood count assessment was performed. Graphs show liver weight/body weight ratio, spleen weight/body weight ratio, and monocyte number. *B*: BM from ribs was collected as described in materials and methods. BM cells were analyzed via flow cytometry for expression of CD163 with exclusion of dead cells using SYTOX blue. Representative flow cytometry plots are shown. *C*: formalin-fixed liver and spleen tissue was prepared and stained for CD163. Representative images are shown.

Weaner pigs were treated with a higher dose of 1.0 mg/kg CSF1-Fc for two daily injections, immediately prior to weaning and on the day of weaning followed by euthanasia 24 h following the final injection. At this early time point, there was already a significant increase in the spleen/body weight ratio and a trend toward increased liver/body weight ratio (Fig. 10*A*). The number of CD163^+^ cells was more than tripled in the bone marrow, from ∼10% to over 30% (Fig. 10*B*), and increased numbers of CD163^+^ macrophages were confirmed by immunostaining in liver and spleen (Fig. 10*C*). At this time point, there was not a significant monocytosis, indicating that both marrow expansion and tissue macrophage proliferation precede monocyte expansion and are likely direct effects of CSF1-Fc.

**Fig. 11.**
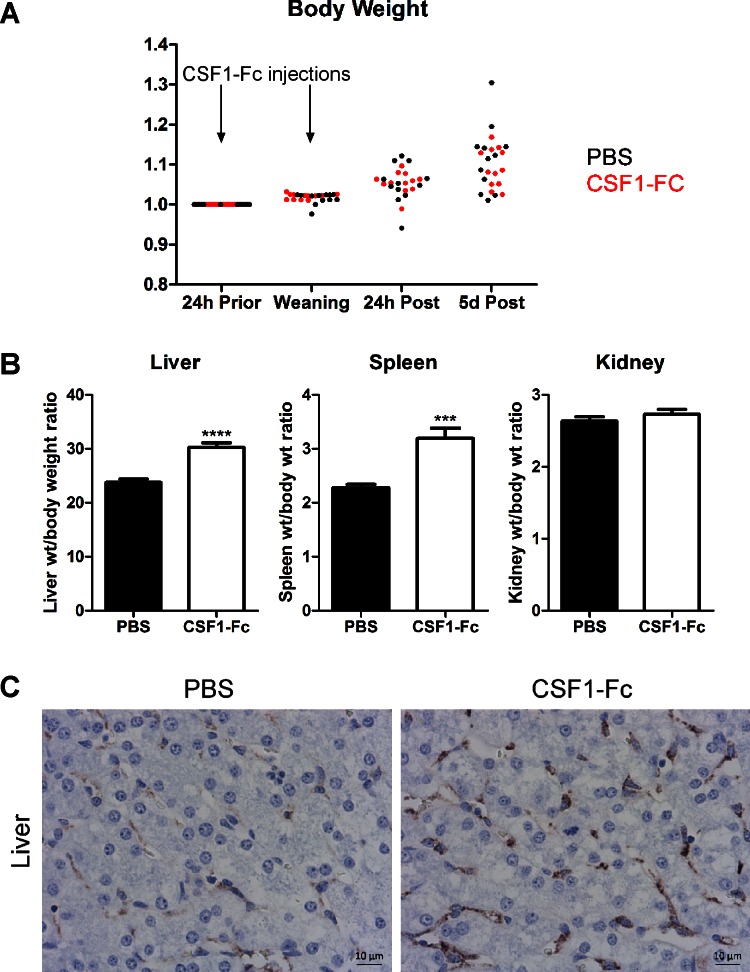
Long-lasting effect of CSF1-Fc in weaning pigs. Pigs (4-wk-old males and females) were injected with PBS or 0.75 mg/kg CSF1-Fc for 2 days prior to euthanasia 5 days after the final injection; *n* = 12 pigs per treatment. *A*: body weight was recorded at each of the time points shown and total body weight change over the duration of the experiment was graphed for PBS treated pigs (black) and CSF1-Fc-treated pigs (red). *B*: bar graphs show means ± SE. ***P < 0.001, ****P < 0.0001 by *t*-test. Graphs show liver weight/body weight ratio, spleen weight/body weight ratio, and kidney weight/body weight ratio. *C*: formalin-fixed liver tissue was prepared and stained for CD163. Representative images are shown. Scale bar = 10 μM.

We repeated the treatment in a larger cohort of weaned pigs. This study was conducted in a high health status research unit, which reflected commercial practice. We explicitly removed zinc from the feed, which is usually added to reduce weaning-associated infections. Given the production of inflammatory cytokines and reduction in IGF-1 in the liver of treated pigs, we measured weight gain daily in all animals. CSF1-Fc (0.75 mg/kg) was administered to pigs for two daily intramuscular injections on the day before and the day of weaning, and pigs were killed 5 days after the second injection. Although some pigs showed evidence of mild postweaning diarrhea, all the animals in both groups continued to gain weight rapidly (Fig. 11*A*). The treated pigs, like the treated mice left for longer after the final injection, demonstrated hepatosplenomegaly (Fig. 11*B*), and the increased numbers of CD163^+^ macrophages in the liver remained evident after 5 days (Fig. 11*C*).

## DISCUSSION

In this study we have extended previous studies in mice (21) to examine the impact of a sustained increase in CSF1 activity on monocyte-macrophage homeostasis. All of the impacts we have observed are consistent with the known biological activity of CSF1. In mice, the same impacts on monocyte-macrophage numbers and maturation can be generated by injection of very much higher doses of native CSF1 (21) or injection of a much larger native form of human CSF1 (24). The doses of native CSF1 required are prohibitive in a large animal. Although we cannot entirely eliminate other functional contributions of the Fc component, the increase in circulating half-life is the most obvious explanation for the increased efficacy compared with native CSF1.

The nature of the so-called hepatostat, which determines that the liver returns to a size that is strictly proportional to body size, has continued to be something of a mystery (34, 35). Although there are many candidates, including growth factors and inhibitors, extracellular matrix proteins and metabolites, and circulating hormones that can regulate hepatic regeneration, it is unclear how any of them functions as a sensor. In a previous study, we made the striking observation that CSF1 treatment of mice (using an Fc conjugate with an increased circulating half-life) was able to increase the size of the liver as well as the number of Kupffer cells. This ability is quite unique. In the present study, we have extended the finding to the domestic pig, an animal that is considerably more human-like in size and vascular biology. The data in Fig. 6 show that a major impact of CSF1-Fc treatment in pigs is to increase the number of hepatocytes through extensive proliferation, so that the total cellularity of the liver is increased even more than the increase in total liver weight. Hepatocyte proliferation, as opposed to hypertrophy, is also a feature of liver regeneration in response to partial hepatectomy (34–36). We have made the reciprocal observation in mice; namely, that prolonged depletion of Kupffer cells with anti-CSF1R treatment leads to a reduction in the size of the liver (45). Others have shown that liver regeneration is greatly impaired in CSF1-deficient or anti-CSF1R-treated mice (46) and in mice depleted of blood monocytes (15) and have promoted liver repair by infusing CSF1-stimulated macrophages into the portal vein (47). The impact of CSF1-Fc on hepatic growth in mice was dependent on monocyte recruitment, as evident from the impact of knockout of CCR2 (46). The role of monocyte-macrophage products, including the inflammatory cytokines TNF, IL1, and IL6, in hepatocyte proliferation has been well recognized (22, 46). CSF1-Fc action in mice was partly dependent on IL6 (46), which was also induced in all of the CSF1-Fc-treated pigs. The effect of CSF1-Fc treatment demonstrates that CSF1-dependent monocyte recruitment is both necessary and sufficient to drive hepatic proliferation and can drive it beyond the homeostatic limits even in a large animal.

The effect of CSF1 treatment supports other evidence of the existence of a homeostatic feedback loop. Macrophages, notably those of the liver (4) and blood monocytes (51), together regulate the level of circulating CSF1 via receptor-mediated endocytosis. This mechanism is evident from the massive increase in circulating CSF1 seen in animals treated with anti-CSF1R (33), and the importance of the liver is evident in patients following partial resection (46). The CSF1-Fc treatment reveals that elevated CSF1 can provoke expansion of the committed monocyte pool in the marrow (Fig. 3), maturation of the monocytes toward a resident phenotype (Fig. 2), and infiltration of the liver (Fig. 5). Hence, the physiological hepatostat (34–36) may actually be a “macrostat.” Of course, the increased macrophage numbers in the liver elicited by CSF1 produce secondary impacts, not only removing potential toxins from the portal blood, but altering the balance of metabolism in the liver between portal and venous-associated functions. Interestingly, resident Kupffer cells are selectively located toward the portal vein in mouse liver lobules (13), which might also serve to localize macrophage-derived hepatocyte proliferative signals.

The expression profiling of the livers of the CSF1-Fc-treated pigs (Fig. 9) closely parallels results obtained previously in the mouse system (21). The newly recruited monocytes clearly respond to TLR-mediated and other signals to express the large majority of transcripts seen when CSF1-stimulated bone marrow-derived macrophages respond to LPS (29). However, classical neutrophil chemoattractants such as IL8 were not detected; we found no evidence of neutrophil infiltration, no induction of classical acute phase response or apoptosis genes, and no evidence of damage to liver cells. Furthermore, the pigs showed no adverse impacts, and in weaners the treatment did not impair their rapid growth (Fig. 11). This is consistent with earlier data, in which recombinant CSF1 has previously been administered in Phase 1 clinical trials by continuous infusion to humans and was well-tolerated (11, 28), and indicates that the Fc fusion protein does not produce any additional toxicity. The lack of severe consequences may be attributed in part to induction of the anti-inflammatory cytokines IL10 and TGFB1 in the liver of the treated animals. Whatever the mechanism, the outcome suggests that CSF1-Fc specifically promotes a proregenerative cellular response in liver, as it does in other organs (23).

In the final set of experiments (Fig. 11), we progressed toward the clinical application of CSF1-Fc in a pig model. The model may also reflect a practical application in pig production to bolster the innate immune system at weaning. We showed that only two doses, administered intramuscularly, were sufficient to produce a sustained increase in monocyte count, liver size, and liver macrophage numbers and produced no adverse local reaction. We propose that CSF1-Fc could provide protection against disseminated infections arising from the immature gut of early-weaned animals. Similarly, clearance functions of the macrophages of the liver are crucial to prevent sepsis in acute liver failure in humans, and CSF1-Fc rapidly promoted clearance functions in mouse disease models (46). The fact that administration of CSF1-Fc to pigs increased the size of the liver and the liver macrophage population (i.e., the clearance capacity, noting also the increased expression of clearance receptors in the array profiles) has an obvious relevance to human acute liver failure. Accordingly, we suggest that the fundamental understanding of the central role of CSF1 in liver homeostasis can potentially translate into both human clinical and veterinary applications.

## GRANTS

This work was funded by Department of Agriculture and Rural Development Evidence and Innovation Fund, project 13/1/04 (E. Magawan). This work was supported by project (BB/J014672/1 and BB/M024288/1) from the Biotechnology and Biological Sciences Research Council (N. A. Mabbott).

The Roslin Institute is supported by Institute Strategic Programme Grant funding and National Capability funding from the Biotechnology and Biological Sciences Research Council (BB/J004235/1, BB/J004316/1, BB/J004227/1, BB/J004332/1, BB/J004243/1).

## DISCLOSURES

D. A. Hume has a patent on CSF1-Fc in liver.

## AUTHOR CONTRIBUTIONS

K.A.S., L.A.W., and D.A.H. conception and design of research; K.A.S., L.A.W., Z.M.L., R.Y., L.L., G.M.D., S.M.C., M.M., and E.M. performed experiments; K.A.S., L.A.W., N.A.M., K.M.S., and D.A.H. analyzed data; K.A.S., L.A.W., N.A.M., K.M.S., and D.A.H. interpreted results of experiments; K.A.S., L.A.W., N.A.M., and K.M.S. prepared figures; K.A.S., L.A.W., and D.A.H. drafted manuscript; K.A.S., L.A.W., N.A.M., K.M.S., and D.A.H. edited and revised manuscript; K.A.S., L.A.W., Z.M.L., R.Y., L.L., G.M.D., S.M.C., M.M., E.M., N.A.M., K.M.S., and D.A.H. approved final version of manuscript.

## Supplementary Material

Supplemental Table 1

Supplemental Table 2
